# The Impact of Hearing Impairment on Patient Care and Autonomy

**DOI:** 10.7759/cureus.65464

**Published:** 2024-07-26

**Authors:** Savannah Baimbridge, Julie Neil, Gabriel M Aisenberg

**Affiliations:** 1 Internal Medicine, University of Texas Health Science Center at Houston - McGovern Medical School, Houston, USA

**Keywords:** geriatrics and internal medicine, altered mental status evaluation, comprehensive geriatric assessment, patient autonomy, hearing impairment

## Abstract

Sensory deficits, including hypoacusis, can cause a barrier to communication between healthcare providers and patients, which in turn can lead to misdiagnosis and loss of patient autonomy. Such deficits are frequently overlooked in clinical encounters. We present a 92-year-old Spanish-speaking female who presented twice to the Emergency Department for complications of a diabetic foot infection. Limited evaluation, documentation, and accommodations regarding the patient’s hypoacusis led to a misinterpretation of her mental status and a transfer of decision-making to surrogates. A two-toe amputation, mechanical intubation, and intensive care unit stay were followed. It was only after these events that the caregivers realized the patient's hypoacusis and learned about her different wishes focused on pain control and hospice care rather than surgical intervention. Available geriatric tools, a consultation with a geriatrician, a thorough evaluation of sensory deficits, and a multidimensional and comprehensive approach could have prevented the loss of autonomy and unexpected care.

## Introduction

Altered mental status (AMS) is a common chief complaint in healthcare, but the indistinct nature of this term can lead to misconceptions of its true etiology. In healthcare, AMS is used for a wide array of symptoms, including changes in attention, cognition, orientation, and arousal [1]. Patients can be labeled with AMS as the broad term described above, but the connotation associated with the term can lead to delay of care, lack of full exploration into alternative etiologies, or inappropriate deference of medical decision-making if the patient is thought to lack capacity.

According to the CDC, 13% of all US adults have reported difficulty hearing [2]. In the population over the age of 65, this number sharply increases to 26.8% [2]. While there is ample literature regarding comprehensive and multidimensional care for older adults, there is limited literature regarding how sensory alterations, such as hearing loss, can affect their care and communication with healthcare providers. This case describes a patient who was labeled as having AMS; two admissions later, including bodily amputations, mechanical ventilation, and an intensive care unit (ICU) admission, all agreed upon by her family members, it was discovered that she had hypoacusis, and should she had been given an opportunity by using the right accommodations, she would have chosen a more conservative path. Thus, this case report demonstrates the importance of using a comprehensive and multidimensional approach in the geriatric population to encompass all possible etiologies of the presenting diagnosis and offer patients autonomy in their care.

## Case presentation

A 92-year-old Spanish-speaking female with a past medical history of type 2 diabetes mellitus, hypertension, and peripheral artery disease presented to the emergency department and was admitted twice to the same facility in the summer of 2023. While the patient’s records revealed no assessment of her hearing ability, during the second admission, the patient was diagnosed with AMS.

During the first admission, the patient presented with a necrotic right second toe and cellulitis extending to her forefoot. The treating physicians resorted to family members when decisions regarding the need for amputation or other aspects of her health were needed. Her second and third right toes were amputated, her postoperative course was uneventful, and the patient was discharged with antibiotics and hydrocodone-based analgesics.

Five days following discharge, the patient was seen in a clinic, where the patient’s family member stated that she had been drowsy and experiencing visual hallucinations, leading to the discontinuation of the opioids three days prior to the clinic visit. The patient was transferred to the emergency department, where she was reportedly alert and oriented but occasionally confused, answering questions inappropriately. The initial admission diagnosis was AMS, which was attributed to infection, hydrocodone, or the progression of underlying dementia. Shortly after admission, she developed increasing oxygen requirements and hemodynamic instability, requiring intubation and pressors in the ICU. Two days later, and after clinical improvement, she was extubated and taken off sedation. After the patient was transferred to the wards, she was awake but was not interacting with the staff. In subsequent conversations, the patient pointed toward her ears, and it became clear that she could not hear. Arrangements for an in-person interpreter with an amplifier device were made. It was then discovered that she was oriented and at baseline mental status, but she was unsure about her diagnosis and management going forward. Proper documentation on the patient’s need for a hearing device and its eventual use allowed the patient to express her wishes for end-of-life care, transitioning to hospice care at home.

## Discussion

This case offers an opportunity to demonstrate one of the barriers that the geriatric population may face in receiving health care. It is understood that the geriatric population has specific considerations in care, but the unique challenges associated with sensory deficits are not well documented in the literature. In this case, the patient’s hearing difficulty potentially caused a loss of patient autonomy and a prolonged diagnosis of AMS that altered the trajectory of her care and was only overcome by a comprehensive care team and a hearing amplifier.

In a two-patient case report, erratic behavior and psychotic features were described, assessed, and treated until appropriate American Sign Language (ASL) interpretations were offered, fully modifying the interpretation of their pictures. These patients displayed coherent thought processes and were using sign language to communicate due to sensory deficits [3]. The impact of verbal language barriers on examining mental status is described in the literature, but the deaf and hard-of-hearing populations are not well represented [4]. With the percentage of older adults affected by hearing loss, it is imperative that providers are aware of and able to recognize this potential confounder to accurately assess mental status. It is also important when considering consent for procedures and participation in decision-making. If a patient is thought to lack capacity, decision-making can be deferred to a family member and disrupt the patient’s autonomy. Awareness of hearing impairment helps prevent these miscommunications in care in the geriatric and hearing-impaired communities.

The use of a multidimensional approach, such as the Comprehensive Geriatric Assessment, offers a potential resource to limit these confounders when working with the geriatric population [5]. Older adults often have many comorbid health conditions that impact their health and the treatment they receive. These conditions, including sensory deficits, might not be as easily recognized through the system-based approach to problems that is normally used in healthcare settings [5]. Thus, utilizing the Comprehensive Geriatric Assessment and increasing awareness of special considerations in the older population can lead to better care, treatment options, and the prevention of complications.

While multiple interventions can address patients’ hearing capacity, the use of such interventions still requires that healthcare practitioners keep the possibility of hypoacusis in their minds and address that diagnosis before assuming the existence of cognitive impairment. No formal algorithm exists in this regard, but the finger rub test, the whispered voice test, the humming test, and formal audiometry have adequate diagnostic accuracy [6-8].

## Conclusions

Caring for a geriatric patient poses a unique challenge due to the various conditions that can contribute to their presentation outside the primary medical concern. Hearing loss, which can be difficult to identify and thus often overlooked, can be confused with AMS. With a multidisciplinary approach and a comprehensive geriatric assessment, potential communication barriers and misdiagnoses can be minimized, supporting greater autonomy.
